# Abstracts

**DOI:** 10.1155/S1463924687000014

**Published:** 1987


Journal of Automatic Chemistry/*Journal of Clinical Laboratory Automation*, Vol. 9, No. 1 (January–March 1987), pp. 1–5

### Abstracts

### Page 6

### Element-specific detectors for high pressure liquid chromatography (HPLC): a computerized, automated HPLC-graphite furnace atomic absorption system

S. J. Haswell *et al.*

Many trace element compounds of biological interest are non-volatile and cannot be identified and quantitatively determined by gas chromatographic techniques. Liquid chromatography is the best technique for the separation of such compounds. The identification of trace element compounds in complex matrices can be easily accomplished with a graphite furnace atomic absorption spectrometer (GFAAS) as an element-specific detector. To join a liquid chromatograph (LC) with the GFAAS, an interface is needed that makes the discontinuously operating GFAAS compatible with the continuous flow of eluate from the column. Such an interface was constructed for a Waters high pressure liquid chromatograph (HPLC) and an Instrument Laboratory Model IL-555/51 GFAAS. The low-cost, computerized interface is easy to construct. It consists of a sampling system taking aliquots from the effluent, an injection device delivering the aliquots to the graphite furnace, and control, timing, and data acquisition circuitry. The HPLC-GFAAS system is controlled by a microcomputer, for which appropriate software was written. The system can analyse an aliquot every 50 s. The results are printed in tabular form providing retention times and signal intensities. Mixtures of arsenic compounds (arsenite, methylarsonic acid, dimethylarsinic acid) were separated.

### Détecteurs spécifiques d'éléments pour la chromatographie liquide à haute pression (HPLC): Un système HPLC à absorption atomique à four de graphique automatisé et informatisé

S. J. Haswell *et al.*

Plusieurs composés d'éléments-traceur d'intérêt biologique sont non-volatiles et ne peuvent être identifiés et determinés quantitativement par les techniques de chromatographie gazeuse. La chromatographie liquide est la meilleure technique pour la séparation de tels composés. L'identification d'éléments-traceur dans des matrices complexes peut parfaitement être effectuée avec un spectromètre d'absorption atomique à four de graphite (GFAAS) en tant que détecteur spécifique d'éléments. Pour relier un chromatographe liquide (LC) avec un spectromètre d'absorption atomique à four de graphite (GFAAS), une interface, rendant compatible le fonctionnement discontinu du GFAAS avec le flux continu de l'éluant provenant de la colonne, est nécessaire. Une telle interface a été construite pour un chromatographe liquide à haute pression (HPLC) de type Waters et pour un spectromètre GFAAS de type Instrument Laboratory Model IL-555/51. L'interface informatisée bon marché est facile à construire. Elle est composée d'un système d'échantillonnage prenant des parts aliquotes de l'effluent, d'un dispositif d'injection transférant les parts aliquotes dans le four de graphite, et de circuits de contrôle, de commande et d'acquisition de données. Le système HPLC-GFAAS est contrôlé par un micro-ordinateur, pour lequel un logiciel approprié a été écrit. Le système peut analyser une part aliquote toutes les cinquante secondes. Les résultats sont imprimés sous forme tabulaire, ils fournissent les temps de rétention ainsi que l'intensité du signal. Des mélanges de composés d'arsenic (arsenite, acide méthylarsonique, acide diméthylarsinique) ont été séparés.

### Elemente-spezifische Detektoren für die Hockdruck-Flüssigchromatographie (HPLC): Ein computerisiertes und automatisiertes HPLC-System mit Graphitofen-Atomabsorptions-Detektion

S. J. Haswell *et al.*

Viele Spurenelement-Verbindungen von biologischem Interesse sind nicht flüchtig und können nicht mit Gaschromatographie identifiziert und quantitativ bestimmt werden. Flüssigchromatographie ist die beste Technik für die Trennung solcher Verbindungen. Die Identifikation von Spurenelement-Verbindungen in komplexen Matrizen kann leicht mit einem Graphitofen-Atomabsorptionsspektrometer (GFAAS) als Elemente-spezifischer Detektor durchgeführt werden. Um einen Flüssigchromatographen (LC) mit einem GFAAS zu verbinden, braucht es ein Schnittstellengerät, das das diskontinuierlich arbeitende GFAAS kompatibel mit dem kontinuierlichen Fluss des Eluats der Kolonne macht. Solch ein Schnittstellengerät wurde für einen Waters Hochdruck-Flüssigchromatographen (HPLC) und ein Instrument Laboratory Modell IL-551/51 GFAAS konstruiert. Das kostengünstige computerisierte Schnittstellengerät ist leicht zu konstruieren. Es besteht aus einem Probennahmesystem, das dem Eluat aliquote Teile entnimmt, ein Injektionssystem, das die aliquoten Teile in den Graphitofen einspritzt und Steuer- und Datenerfassungsschaltungen. Das HPLC-GRAAS wird durch einen Mikrocomputer gesteuert, für den entsprechende Software geschrieben wurde. Das System kann alle fünfzig Sekunden ein Aliquat analysieren. Die Resultate werden in einer Tabelle, enthaltend die Retentionszeit und die Signalintensität, ausgedruckt. Mischungen von Arsenverbindungen (Arsenit, Methylarsonsäure, Dimethylarsensäure) wurden getrennt.

### Page 15

### A versatile computerized polargraph, polarographic sample changer and data acquisition system: applications in electrode mechanism studies

H. H. J. L. Ploegmakers *et al.*

A fully computerized polarograph, polarographic sample changer and data-acquisition system is described. The system consists of a home-made sample changer, commonly available computer hardware and home-made software. The system performs automatically sampled direct current (DC) polargraphy of a series of a maximum of 40 samples. The half-wave potential(s) and the limiting current(s) of the thus obtained polarographic curves can be automatically calculated, for example to analyse electrochemical parameters of a compound at (40) different pH-values. The relationship of these parameters versus pH can be easily and automatically determined. The system was developed for computerized polarographic analysis of anticancer drugs, but it can be applied to the analysis of any other organic or inorganic compound. Important features of the system are: automatic sample changing, pH measurement of the polarographic solutions, blank value subtraction, half-wave potential and limiting current calculation, logarithmic analysis of polarographic waves and examining pH-dependencies of electrochemical parameters, plotting half-wave potential and limiting current versus pH and calculating and plotting of the slope of the half wave potential-pH relationship.

To show the applicability of the system, the analysis of the electrode mechanism of 2-amino-1,4-naphtoquinone and 2,5-bis(1-aziridinyl)-1,4-benzoquinone has been presented, as part of a study to determine the *in vivo* redox activation mechanism of quinones as anticancer drugs.

### Un polarographe informatisé à usage multiple, un changeur d'échantillons polarographiques et un système d'acquisition de données: Applications à l'étude du mécanisme des électrodes

H. H. J. L. Ploegmakers *et al.*

Un polarographe complétement informatisé, un changeur d'échantillons polarimé triques et un système d'acquisition de données sont décrits. Ce système est composé d'un changeur d'échantillons fait-maison, d'un matériel électronique d'ordinateur communément disponible, ainsi que de logiciel fait-maison. Le système effectue une analyse polarographique à courant continu de façon automatique sur une série de 40 échantillons au maximum. Le(s) potentiel(s) demi -onde et le(s) courant(s) limite(s) de polarogrammes ainsi obtenus, peuvent être calculés automatiquement, par exemple lors de l'analyse des paramètres électrochimiques d'un composé pour 40 valeurs différentes du pH. La relation entre ces paramètres et le pH peut facilement être déterminéee automatiquement. Le système a été développé pour une analyse polarimétrique informatisé de drogues anticancéreuses, mais il peut être appliqué pour l'analyse de tout autre composé organique ou inorganique. Les caractéristiques importantes du système sont: le changement automatique de l'échantillon, la mesure du pH des solutions polarographiques, la soustraction de la valeur à blanc, le calcul du potentiel demi-onde et du courant limite, l'analyse logarithmique des ondes polarimétriques et l'examen de la dépendance en pH des paramètres électrochimiques, le traçage graphique du potentiel demi-onde et du courant limite en fonction du pH, et enfin, le calcul et le traçage graphique de la pente de la relation entre le potential demi-onde et le pH.

Pour montrer l'applicabilité de ce système, l'analyse du mécanisme d'électrode de 2-amino-1,4-naphtoquinone et de 2,5-bis(1-aziridinyl)-1,4-benzoquinone a été presentée, en tant que partie d'une étude consacrée à la détermination in-vivo du mécanisme d'activation redox de quinones utilisées comme drogues anticancéreuses.

### Ein vielseitiger, computerisierter Polarograph, polarographischer Probenwechsler und Datenerfassungssystem: Anwendungen in Studien der Elektrodenmechanismen

H. H. J. L. Ploegmakers *et al.*

Ein voll computerisierter Polarograph, ein polarographischer Probenwechsler und ein Datenerfassungssystem werden beschrieben. Das System besteht aus einem selbstgebauten Probenwechsler, überall erhältlicher Computer-Hardware, und selbstgeschriebener Software. Das System führt Gleichstrompolarographie an Serien mit maximal 40 Proben unter automatischer Probenahme durch. Die Halbwellen Potentiale und die Grenzströme der so erhaltenen Polarogramme können automatisch verwendet werden, z.B. um elektrochemische Parameter einer Verbindung bei 40 verschiedenen pH-Werten zu analysieren. Die Relation zwischen diesen Parametern und dem pH kann leicht und automatisch bestimmt werden. Das System wurde für die computerisierte polarographische Analyse von Krebsheilmitteln entwickelt. Es kann aber für die Analyse jeder anderen organischen oder anorganischen Verbindung eingesetzt werden. Wichtige Eigenschaften des Systems sind: Automatischer Probenwechsler, pH-Messung der polarographischen Lösung, Leerwert-Korrektur, Halbwellenpotential- und Grenzstrom-Berechnung, logarithmische Analyse der polarographischen Wellen und Untersuchung der pH-Abhängigkeit elektrochemischer Parameter, grafische Aufzeichnung der Halbwellenpotentiale und Grenzströme in Funktion des pH, und Berechnung und Aufzeichnung der Steigerung der Halbwellenpotential-pH Relation.

Um die Anwendbarkeit des Systems zu zeigen, wird die Analyse des Elektrodenmechanismus von 2-Aminol,4-Naphtochinon und 2,5-bis(1-Azaridinyl)-1,4-Benzochinon vorgestellt, die Teil einer Studie zur Bestimmung der in vivo Redoxaktivierungs-mechanismen von Chinonen als Krebsheilmittel sind.

---

### Page 25

### The systematic error caused by random errors through data reduction

K. M. Hangos and L. Leisztner

Data reduction transformation is used for producing analytical information from the discretized measurement signal. Through mathematical analysis of the data reduction transformation it was shown (for non-linear transformations) that the random error in signal samples is transformed into systematic error in the analytical information.

In evaluations of chromatograms the individual steps of data reduction, peak recognition, nonlinear base-line correction and peak height calculation proved to be nonlinear transformations.

The magnitude and sign of the systematic error caused by data reduction during the evaluation of chromatograms was investigated by simulation experiments as a function of the signal-to-noise ratio and the peak shape.

*French and German abstracts will follow in the April issue.*

---

### Page 30

### Role of valves in non-segmented flow systems

A. Ríos *et al.*

This paper shows the significance of the valves used in non-segmented flow systems, especially in Flow Injection Analysis, considered as a means of inserting the sample into the system as well as their role in the generic manipulation of the configuration. The different types of injection devices (rotary valves, proportional injectors etc.) and selecting and diverting valves (both commutator and rotary), as well as their different possible arrangements in flow systems are considered.

*French and German abstracts will follow in the April issue.*

---

### Page 37

### Analysing the effects of hardware upgrades on the performance of a LABCOM+ clinical laboratory computer system

Arthur A. Eggert *et al.*

The change in performance that occurs when a laboratory computer system is upgraded in hardware is important to measure because it gives tangible proof as to whether the upgrade accomplished the stated goals and whether the cost-to-benefit ratio was excessive. While vendors may give glowing estimates of performance improvement, these are very specific to the type of system load used in the benchmarking procedure and are not always accurate in predicting what will happen in a clinical laboratory environment. Benchmarking procedures for clinical laboratory systems have been generally unavailable. The authors present a procedure for benchmarking systems before and after upgrades and show the degree of improvement that can be obtained by replacing a central processing unit and by optimizing data storage among different storage devices. Improvements are tied to the disk-wait/compute-time ratio and the average system utilization.

---

### Page 41

### Artifactually elevated BUN values on the Boehringer Mannheim-Hitachi 737 and 705 automated chemistry analysers and the development of a kinetic BUN method

William E. Neeley *et al.*

Serum analysis using enzymatic reactions with Boehringer Mannheim's Hitachi 737 and 705 instruments may yield falsely elevated BUN values for some patients. This investigation has indicated that these false values occurred when a patient's serum contained abnormal levels of a monoclonal gamma globulin. In each of three cases discovered, the patient's serum proteins precipitated in the first reagent and dissolved upon addition of the second reagent. A new reagent system and a kinetic method of analysis have been developed in which these abnormal monoclonal proteins no longer interfere.

*French and German abstracts will be published in the April issue.*

---

### Page 44

### An aid to interfacing analytical instrumentation to laboratory computer systems

Ian Clark and Margaret Peters

A simple microcomputer printer buffer is offered as a low-cost means of interfacing analytical instrumentation and laboratory computer systems.

*French and German abstracts will be published in the April issue.*

---

### Page 46

### Amperometric determination of nitrogen dioxide in air samples by flow injection and reaction at a gas-liquid interface

Frazier W. Nyasulu and Horacio A. Mottola

Continuous-flow processing of air samples for repetitive determinations of $NO_2(g)$ is presented. The determination is based on the $NO_2$ oxidation of tris(1,10-phenanthroline)iron(II) [ferroin], to the iron(III) analogue [ferriin], at a gas-liquid interface. The extent of ferroin oxidation is amperometrically monitored at a carbon paste electrode and the change in current level at 780 mV (versus SCE) is proportional to the $NO_2$ concentration in the air sample. The performance of a gas diffusion flow injection system is compared with that of a direct intercalation procedure of gas sample into a liquid carrier. Determination is possible in the 12 to 140 ppm (v/v) and 0.5 to 11 ppm (v/v) range with the gas diffusion and the direct intercalation procedures, respectively. Typical relative standard deviations are 2 to 3% (10 determinations) and typical sampling rates are 35 h for the gas diffusion system and 25 h with the direct intercalation approach.

*French and German abstracts will be published in the April issue.*

---

*French and German abstracts of papers in Volume 8, Number 4 (October–December 1986).*

### Page 186

### Déterminations cinétiques dans l'analyse d'écoulements continus

M. D. Luque de Castro and M. Valcàrcel

Un aperçu des méthodes cinétiques mises en oeuvre dans des systèmes d'écoulements continus segmentés et non-segmentés est présenté. Les caractéristiques des déterminations cinétiques ainsi qu'une classification des méthodes de mesure de vitesses de réaction et des méthodes cinétiques continues sont discutées.

### Kinetische Bestimmungen in der kontinuierlichen Durchflussanalyse

M. D. Luque de Castro and M. Valcàrcel

Es wird eine Uebersicht der kinetischen Methoden, die auf kontinuierlichen, segmentierten und nicht-segmentierten Systemen realisiert wurden, gegeben. Die Charakterisierung kinetischer Bestimmungen und eine Klassifizierung von Reaktionsgeschwindigkeits-Methoden und kontinuierlichen Kinetik-Methoden sind diskutiert.

---

### Page 197

### Calcium ionisé dans sérum: Evaluation de l'analyseur de calcium Analyte +2 dans un laboratoire de chimie clinique

Rosemarie Freaney *et al.*

Une évaluation du système Analyte +2 utilisé pour mesurer simultané ment l'activité du calcium ionisé et le pH dans du sérum et du sang complet est présentée. La corrélation des résultats concernant le calcium ionisé obtenus avec le système Analyte +2 et un autre analyseur, le système Orion SS 20, a fourni un coefficient de 0·986. La précision de la détermination du calcium ionisé dans du sérum ainsi que dans du matériel de contrôle en solution aqueuse ou dans du sérum avec l'instrument Analyte +2 est donneée. La précision fut a peu près la même, que le calcium ionisé soit corrigé pour un pH de 7.4 ou non. La sélectivité de l'électrode pour le calcium a été étudiée en changeant la concentration de potasssium, de magnesium et de sodium. Les changements de ces ions dans les limites physiologiques ont montré un effet minimal sur le calcium ionisé. L'effet, sur le calcium ionisé et sur le pH, d'un entreposage de l'échantillon de courte ou de longue durée a été évalué. Un changement minimal du pH du sérum s'est manifesté après 4 heures, +0·004 +/0·009 (moyenne +/déviation standard). La diminuation moyenne du calcium ionisé après 2 jours fut de 0·005 +/0·010 mmol/l. On en conclut que les résultats concernant le calcium ionisé, obtenu aprés stockage du sérum dans une seringue à 4 C°, sont cliniquement utilisables. Un domaine de référence pour le calcium ionisé dans le serum, de personnes en bonne santé a été établi et a donné pour valeur moyenne: 1·26 mmol/l. Gamme: (+/2 déviations standard) 1·19 – 1·33 mmol/l.

### Ionisierter Kalzium in Serum: Auswertung des ANALYTE +2 Kalzium-Analysengerätes in einem klinisch-chemischen Laboratorium

Rosemarie Freaney *et al.*

Eine Auswertung des ANALYTE +2 Systems für die gleichzeitige Bestimmung der Kalziumionen-Akivität und des pH in Serum und ganzem Blut wird vorgestellt. Die Korrelation der Resultate ionisierten Kalziums, erhalten mit den ANALYTE +2 und einem anderen Gerät, dem ORION SS20 System, ergab einen Koeffizienten von 0·986. Die Präzision der Bestimmung von ionisiertem Kalzium in Serum und in wässrigen und serumartigen Kontrollmaterialen mit dem ANALYTE +2 werden angeführt. Die Präzision war ähnlich, gleichgültig ob ionisiertes Kalzium unkorrigiert blieb oder auf pH 7·40 korrigiert wurde. Die Selektivität der Elektrode wurde getestet durch die Variation von Kalium, Magnesium und Natrium. Aenderungen im physiologischen Bereich dieser Ionen zeigten eine minimale Wertung auf ionisiertes Kalzium. Die Wertung von Kurz- und Langzeitspeicherung auf ionisertes Kalzium und pH wurde abgeschätzt. Eine minimale Aenderung im Serum pH trat nach 4 Stunden ein, +0·004 +/0·009 (Mittelwert +/Standardabweichung). Die mittlere Abnahme an ionisiertem Kalzium nach 2 Tagen betrug 0·005 +/0·010 mMol/l. Wir schliessen daraus, dass Resultate für ionisiertes Kalzium, erhalten nach Speicherung des Serums in einer Spritze bei 4 °C, klinisch verwendbar sind. Ein Referenzbereich für ionisiertes Kalzium im Serum gesunder Kontrollpersonen wurde etabliert und ergab einen Mittelwert von 1.26 mMol/l. Bereich (+/2 Standardabweichungen) 1·19 – 1·33 mMol/l.

---

### Page 202

### Time utilities: Programme en MUMPS pour le calcul des intervalles de temps et le chronométrage des événements

T. G. Pellar and A. R. Henderson

Le langage MUMPS permet une manipulation aisée des dates et des heures. Nous décrivons un ensemble de quatre programmes écrits en MUMPS. Ces programmes permettent 1) de calculer l'intervalle de temps (en jours ou en heures) séparant des événements séquentiels ou non, 2) de conserver sur des fichiers la chronologie d'événements sur une longue période (p.ex. lors de transplantation), 3) d'utiliser un terminal en tant que temporisateur, et 4) d'utiliser un terminal en tant que générateur d'intervalles de temps. Ces programmes se sont révélés d'une très grande utilité dans un département de biochimie clinique d'un hopital.

---

### Time utilities: Programme in MUMPS für die Berechnung von Zeitintervallen und für die Zeitmessung von Ereignissen

T. G. Pellar and A. R. Henderson

Die Programmiersprache MUMPS erlaubt einfaches Behändigen von Kalender-Daten und Zeiten. Wir beschreiben einen Satz von vier Programmen, geschrieben in MUMPS, die 1. die Zeit (in Tagen oder Stunden) zwischen Ereignissen oder sequentiellen Ereignissen berechnen, 2. Datenmengen von Subjekten in Speichern zurückhalten, für die über eine längere Periode Zeiten gemessen werden (z.B. Transplantations-Patienten), 3. die Verwendung eines Terminals zur Zeitmessung gestatten und 4. die Verwendung eines Terminals als Zeitgeber erlauben. Die Programme haben sich in einer klinisch-biochemischen Spitalabteilung als äusserst nützlich erwiesen.

---

### Page 207

### Evaluation comparative d'un système Technicon SMAC2/RA1000 et d'un analyseur Parallel d'American Monitor sous conditions normales de travail

A. J. Little *et al.*

Pour beaucoup de grands laboratoires de biochimie clinique, le choix d'un analyseur prinicipal se fait entre un système Parallel (American Monitor U.K.) et une combinaison SMA2/RA1000 (Technicon U.K.). Cette décision est non seulement importante à cause de la somme élevée à investir, mais aussi du fait qu'elle est pratiquement irréversible après installation et acceptation du système. Ainsi, pour opérer ce choix, tous les moyens d'aide à la décision sont rassemblés. Mais, cela est souvent subjectif. Les critères utilisés ne reposent pas toujours sur des faits, ou bien, ne sont pas comparables. Dans le but de fournir l'information nécessaire pour ce choix, nous avons entrepris une évaluation comparative de ces deux systémes sous conditions normales de travail. La puissance d'analyse, la fiabilité et les coûts d'exploitation ont été examinés et evalués durant une période de 15 jours. Cette étude a montré que la combinaison SMAC2/RA1000 est plus fiable et moins imprécise que le système Parallel. En ce qui concerne les coûts relatifs, on peut conclure qu'ils sont en accord avec les exigences et la configuration de ces deux systèmes, qui utilisent des concepts très différents pour manipuler et analyser les échantillons.

**Vergleichende Evaluation eines Technicon SMAC2/RA1000 Systems und eines Parallel von American Monitor während des normalen Dienstleistungsbetriebs**
A. J. Little *et al.*

Die Wahl eines Haupt-Analysengerätes besteht für viele grosse klinische Laboratorien zwischen einem Parallel (American Monitor U.K.) und einer SMAC2/RA1000 Kombination (Technicon U.K.). Das ist nicht nur wegen der Grösse der Investition ein wichtiger Entscheid, sondern er ist normalerweise auch irreversibel nach Installation und Abnahme. Deshalb wird für den Entscheid jede verfügbare Hilfe mobilisiert. Jedoch ist diese häufig anekdotenhaft als auf Tatsachen beruhend und üblicherweise auch nicht vergleichender Natur. Um solche Informationen zur Verfügung zu stellen, haben wir eine vergleichende Evaluation dieser beiden Systeme unter Routinebedingungen durchgeführt. Analytische Leistung, Zuverlässigkeit und Betriebskosten wurden während 15 Wochen verfolgt. Die Studie ergab, dass die SMAC2/RA1000-Kombination zuverlässiger und weniger ungenau ist als das Parallel Analysengerät. Schlussfolgerungen in bezug auf die relativen Kosten stehen in Uebereinstimmung mit den Anforderungen und Umständen; die beiden Systeme verwenden indessen stark verschiedene Konzepte für die Probenbearbeitung und die Analyse.

### FORTHCOMING PAPERS

**The use of queueing theory for planning automated analytical systems**
*T. L. Pap and L. Leisztner*

**Automated, quantitative, low-pressure, cation-exchange chromatography of haemoglobin variants on midget columns**
*R. S. Ersser, R. E. Drew, G. B. Barlow and M. Hjelm*

A computer-controlled prototype system for automated, quantitative, liquid chromatographic separation of mixtures of human haemoglobins is described. Cation-exchange chromatography was performed on a 5 × 4·5 mm midget column, filled with 10 µm diameter spheres of porous (300 A) silica, coated with polyaspartic acid. The column was eluted, using a low-pressure peristaltic pump, with gradients of sodium ions in a bis-tris buffer (40 mM, pH 6·2).

Gradient profiles suitable for general haemoglobin studies, glycated haemoglobin and neonatal screening for sickle-cell disease have been devised. The precision and accuracy of these methods was investigated. Peak elution times, between batch, had a coefficient of variation (CV) less than 0·8%, peak area versus amount of haemoglobin loaded was linear between 30–350 µg, and the percentage total for haemoglobins, between batch, had CVs less than 5%.

**Comparison of a time-resolved fluoroimmunoassay with a solid phase immunoradiometric assay for the measurement of alphafetoprotein in amniotic fluid**
*E. J. Coombes, B. J. Moody and H. James*

The analytical and clinical performance of a time-resolved fluoroimmunoassay (dissociation enhanced lanthanide fluoroimmuno assay, DELFIA, LKB Wallac) has been compared with a solid phase immunoradiometric assay (IRMA, Boots Celltech Diagnostics Ltd) for the measurement of $\alpha$ fetoprotein (AFP) in amniotic fluid. Comparison of results using 174 amniotic fluids (115 normals, 55 cases of open neural tube defects and four amniotic fluids with equivocal results) gave an excellent correlation ($r$ = 0·997: IRMA = 1·155 DELFIA + 0·946). Comparison of the analytical performance of the two assays, as assessed by intra- and inter-assay coefficients of variation, gave similar results over a concentration range of 27·5 to 183·9 MU/l. Furthermore, the diagnostic performance of the DELFIA in predicting proven fetal abnormalities agreed exactly with the IRMA.

